# Gastrointestinal Complications Associated With Non-steroidal Anti-inflammatory Drug Use Among Adults: A Retrospective, Single-Center Study

**DOI:** 10.7759/cureus.26154

**Published:** 2022-06-21

**Authors:** Nouf Alhammadi, Abdullah H Asiri, Fatimah M Alshahrani, Alhanouf Y Alqahtani, Maraam M Al Qout, Raad A Alnami, Ahlam S Alasiri, Ahmed S AL-Zomia

**Affiliations:** 1 Internal Medicine, King Khalid University, Abha, SAU; 2 Medicine and Surgery, King Khalid University, Abha, SAU; 3 College of Medicine, King Khalid University, Abha, SAU

**Keywords:** steroid use, population, saudi arabia, risk factors, gastrointestinal complications, gastric mucosal hemorrhages, injury git mucosa, git complications, nsaid

## Abstract

Background

Traditional non-steroidal anti-inflammatory drugs (NSAIDs) are recognized to injure the upper gastrointestinal tract (GIT) mucosa. For example, gastric mucosal hemorrhages can be caused by a single dose of 650 mg of aspirin. Nearly 30% to 50% of NSAID users showed endoscopic lesions including subepithelial hemorrhages, erosions, and ulcerations. These lesions are often asymptomatic and are mostly found in the gastric antrum. With the chronic exposure, the mucosa adapts to the aggression of the NSAIDs, whereby these lesions slowly lessen or fade.

Aim

The aim of this study is to detect the association between NSAIDs and gastrointestinal complications among the general population in the Aseer region, Saudi Arabia.

Methodology

A record-based retrospective study was conducted targeting people with GIT complications who were 20 years old and above. We reviewed patients' records from the GIT clinic in the Aseer region of Saudi Arabia. We reviewed specifically patients who had GIT complications like gastritis, abdominal pain, GIT bleeding, heartburn, nausea, vomiting, peptic ulcer, and diarrhea. Then we contacted those patients individually to fill out a questionnaire. Participants less than 18 years, those who refused to complete the questionnaire, or any patients with no history of GIT complications were excluded. An online questionnaire was sent to the patients with GIT complications. The questionnaire included participant's personal data, NSAID use, and associated GIT complications. The questionnaire was uploaded online using social media platforms by the researchers and their relatives and friends during the period from March 2012 to May 2022.

Results

A total of 211 participants with GIT complications completed the study questionnaire. Participant ages ranged from 20-59 years with a mean age of 31.2 ± 12.9 years old. A total of 140 (66.4%) were males and 175 (82.9%) were from urban areas. A total of 156 (73.9%) were non-smokers. A total of 103 (48.8%) participants used NSAIDs. As for complications, the most reported were peptic ulcer (37.9%), GIT bleeding (5.8%), GIT erosions (4.9%), and intestinal obstruction (3.9%) while 59.2% had no complications.

Conclusions

The current study revealed that nearly one out of every two participants in the Aseer region mainly used NSAIDs as tablets for pain. Regarding high utilization rates, less than half of them developed GIT complications, mainly peptic ulcers.

## Introduction

Globally, non-steroidal anti-inflammatory drugs (NSAIDs) are among the most frequently prescribed and used medications [1]. NSAIDs have analgesic, antipyretic, or anti-inflammatory uses. A variety of side effects on the gastrointestinal tract are reported among NSAID users [2]. They are given mostly to patients above 65 years old and comprise 8% of medical prescriptions worldwide. Furthermore, the over-the-counter use of NSAIDs is increasing, as 26% of the users are beyond the recommended dose and many are unprescribed [3,4]. Traditional NSAIDs are recognized to injure the upper GIT mucosa. For example, gastric mucosal hemorrhages can be caused by a single dose of 650 mg of aspirin. Furthermore, if the same dose was given four times daily, it will cause gastric erosions [5]. Nearly 30% to 50% of NSAID users showed endoscopic lesions, including subepithelial hemorrhages, erosions, and ulcerations. These lesions are often asymptomatic and mostly found in the gastric antrum. With the chronic exposure, the mucosa adapts to the aggression of the NSAIDs, whereby these lesions will slowly lessen or fade [6-8].

On the other hand, upper gastrointestinal (GI) symptoms appeared among about 40% of NSAID users, with the most frequently diagnosed including gastroesophageal reflux, featured by regurgitation and/or heartburn, and dyspeptic symptoms, including belching, epigastric discomfort, bloating, early satiety, and postprandial nausea [9]. Moreover, lower gastrointestinal tract (GIT) injury can be caused by NSAIDs, where it causes harm below the duodenum, involving the small and large intestines. A nonrandomized trial was conducted on 21 arthritis patients, who were on NSAIDs for more than 3 months, and 20 controls, who had acetaminophen or no analgesics at all. The video capsule endoscopy of 71% of NSAID users and 10% of controls showed some mucosal damage characterized by red spots, erosions, or ulcers. Furthermore, large erosions or ulcers were seen in 23% of NSAID users. The control group had no large erosions and ulcers [5]. The recognition of the GIT effects caused by NSAIDs is crucial for developing better therapeutic plans that focus on the supportive care and prevention of GIT complications and eventual organ failure. The current study aimed to assess the use and risk of gastrointestinal complications related to the use of NSAIDs among the population in the Aseer region in southern Saudi Arabia.

## Materials and methods

A record-based retrospective study was conducted targeting a population with GIT complications in the Aseer region, Saudi Arabia. We reviewed patients' records from the GIT clinic in the Aseer region, Saudi Arabia, specifically records of patients admitted with GIT complications who are 20 years of age and above. Then we contacted those patients individually to fill out a questionnaire. Participants less than 20 years old, those who refused to complete the questionnaire, and patients with no GIT complications were excluded. After obtaining ethical approval, we sent an online questionnaire to the selected population with GIT complications. Around 211 patients with GIT complications responded to the questionnaire. The questionnaire was developed by researchers after intensive literature reviews and expert consultation. The first part of the questionnaire included the participant's personal data, medical and drug history, smoking, and drug allergy. The second part covered the use of NSAIDs, frequency of use, the pattern of use, prescription, and causes of using NSAIDs. The third part covered NSAID use-associated GIT side effects and complications. The questionnaire was uploaded online using social media platforms by the researchers during the period from March 2021 to May 2022.

Data analysis

After data was extracted, it was revised, coded, and fed to statistical software IBM SPSS version 22 (IBM Corp., Armonk, USA). All statistical analysis was done using two-tailed tests. A p-value less than 0.05 was considered statistically significant. Descriptive analysis based on frequency and percent distribution was done for all variables including participants' age, gender, residence, smoking, chronic diseases, and drug use. Also, participants' use of NSAIDs, frequency of use with associated complications, and side effects were tabulated and graphed. Cross-tabulation was used to assess factors associated with NSAID use and related complications among study participants. Relations were tested using the Pearson Chi-square test and the Exact probability test for small frequencies.

## Results

A total of 211 participants completed the study questionnaire. Participant ages ranged from 20-59 years with a mean age of 31.2 ± 12.9 years old. A total of 140 (66.4%) were males and 175 (82.9%) were from urban areas. As for smoking, 156 (73.9%) were non-smokers while 48 (22.7%) were smokers. A total of 68 (32.2%) had a chronic health problem which was for more than 10 years among 44 (64.7%) and for 5-10 years among seven (10.3%). A total of 50 (23.7%) patients had a history of allergy and 57 (27%) used other drugs.

**Figure 1:**
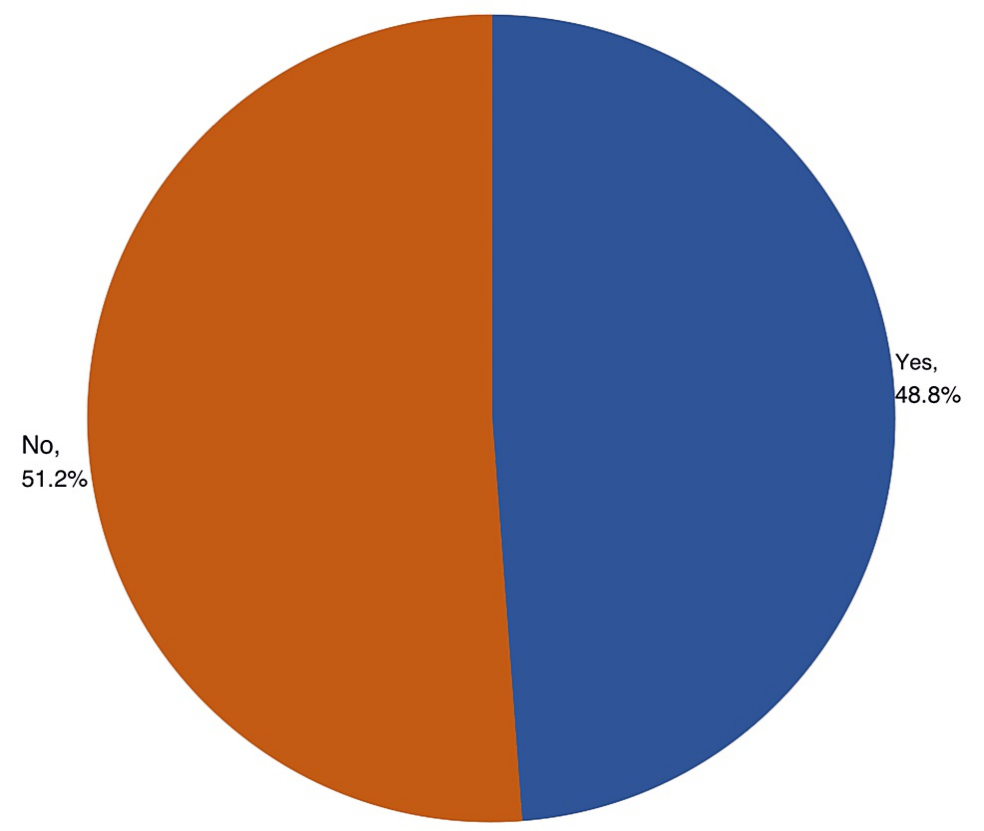
Prevalence of NSAID use among the population with GIT complications, Aseer Region, Saudi Arabia NSAID: non-steroidal anti-inflammatory drugs; GIT: gastrointestinal tract

A total of 103 (48.8%) participants used NSAIDs (Figure 1). Regarding the pattern of use, 72 (69.9%) reported using the drugs with pain, 16 (15.5%) used the drug daily, and 10 (9.7%) with fever. NSAIDs were prescribed by a physician among 71 (68.9%) of the users, by a pharmacist among 13 (12.6%), by a relative/friend among 14 (13.6%), and five had them unprescribed,** **by themselves. A total of 61 (59.2%) NSAID users had the drugs after meals while 26 (25.2%) had before meals. As for the frequency of taking NSAIDs/day, it was taken once daily among 46 (44.7%) and twice-daily among 34 (33%). A total of 84 (81.6%) had the drugs as tablets/capsules (Table 1). 

**Table 1:** Pattern of use of NSAIDs among the population with GIT complications, Aseer Region, Saudi Arabia NSAIDs: non-steroidal anti-inflammatory drugs; GIT: gastrointestinal tract

The pattern of NSAIDs use	No	%
When do you use NSAIDs?		
With pain	72	69.9%
With fever	10	9.7%
With physician prescription	5	4.9%
Daily	16	15.5%
Who prescribed the NSAIDs for you?		
Physician	71	68.9%
Pharmacist	13	12.6%
Relatives/friends	14	13.6%
None	5	4.9%
Time to have NSAIDs		
After meals	61	59.2%
Before meals	26	25.2%
Any time	16	15.5%
Frequency of taking NSAIDs / day		
One time	46	44.7%
Two times	34	33.0%
Three times	16	15.5%
4 / more times	7	6.8%
Formula using NSAIDs		
Tablet / capsule	84	81.6%
Injection	19	18.4%

A total of 80% of participants aged more than 50 years used NSAIDs versus 32.3% of those aged 20-30 years (p=.001). Also, 73.5% of those with chronic health problems used NSAIDs compared to 37.1% of others without (p=.001). NSAIDs were used by 66.7% of used other drugs in comparison to 42.2% of others who did not (p=.002) (Table 2). 

**Table 2:** Factors associated with NSAID use among the population with GIT complications, Aseer region, Saudi Arabia p-value: Pearson's χ2 test; ^$^Exact probability test; *p < 0.05 (significant) NSAID: non-steroidal anti-inflammatory drugs; GIT: gastrointestinal tract

Factors	Total		Use NSAIDs				p-value
			Yes		No		
	No	%	No	%	No	%	
Age in years							.001*
20-30	65	30.8%	21	32.3%	44	67.7%	
30-40	71	33.6%	35	49.3%	36	50.7%	
40-50	45	21.3%	23	51.1%	22	48.9%	
> 50	30	14.2%	24	80.0%	6	20.0%	
Gender							.848
Male	140	66.4%	69	49.3%	71	50.7%	
Female	71	33.6%	34	47.9%	37	52.1%	
Residence							.601
Urban	175	82.9%	84	48.0%	91	52.0%	
Rural	36	17.1%	19	52.8%	17	47.2%	
Smoking							.428^$^
Current smoker	48	22.7%	27	56.3%	21	43.8%	
Ex-smoker	7	3.3%	4	57.1%	3	42.9%	
Non-smoker	156	73.9%	72	46.2%	84	53.8%	
Had chronic health problems							.001*
Yes	68	32.2%	50	73.5%	18	26.5%	
No	143	67.8%	53	37.1%	90	62.9%	
Disease duration							.117^$^
1-2 years	7	10.3%	3	42.9%	4	57.1%	
3-5 years	10	14.7%	6	60.0%	4	40.0%	
5-10 years	7	10.3%	5	71.4%	2	28.6%	
> 10 years	44	64.7%	36	81.8%	8	18.2%	
History of allergy							.270
Yes	50	23.7%	21	42.0%	29	58.0%	
No	161	76.3%	82	50.9%	79	49.1%	
Use other drugs?							.002*
Yes	57	27.0%	38	66.7%	19	33.3%	
No	154	73.0%	65	42.2%	89	57.8%	

**Figure 2:**
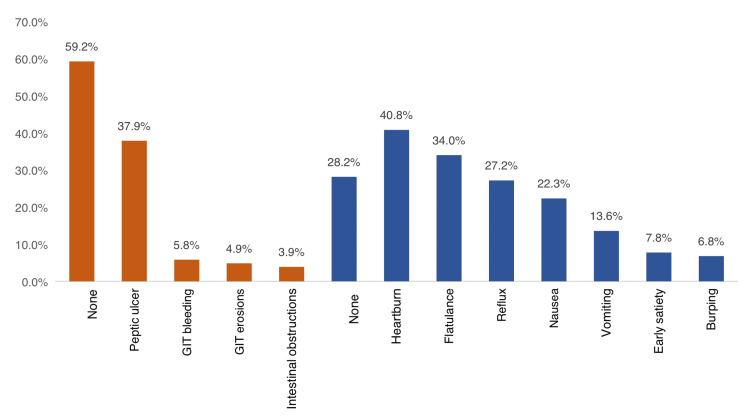
GIT side effects and complications due to the use of NSAIDs, Aseer Region, Saudi Arabia NSAID: non-steroidal anti-inflammatory drugs; GIT: gastrointestinal tract

The most reported side effects among users were heartburn (40.8%), flatulence (34%), reflux (27.2%), nausea (22.3%), vomiting (13.6%), early satiety (7.8%), and burping (6.8%) while 28.2% had no side effects. As for complications, the most reported were peptic ulcer (37.9%), GIT bleeding (5.8%), GIT erosions (4.9%), and intestinal obstruction (3.9%) while 59.2% had no complications (Figure 2). 

The complications were diagnosed with clinical signs and symptoms among 6 (9.5%), by signs/symptoms with lab investigations among 37 (58.7%), while 20 (31.7%) needed endoscopy to diagnose complications. A total of 42 (40.8%) reported still using NSAIDs and 56 (91.8%) of those who stopped using NSAIDs said it was their own decision. 

**Table 3:** NSAIDs-related complications among the population with GIT complications in Aseer region, Saudi Arabia NSAID: non-steroidal anti-inflammatory drugs; GIT: gastrointestinal tract

Complications data	No	%
Method of diagnosing complications		
Clinical signs & symptoms	6	9.5%
Clinical & laboratory investigations	37	58.7%
Endoscopy	20	31.7%
Still using NSAIDs		
Yes	42	40.8%
No	61	59.2%
If stopped, why?		
By physician	5	8.2%
By my self	56	91.8%

A total of 51.9% of the current smokers experienced complications versus 75% of ex-smokers and 34.7% of non-smokers (p=.049). Also, 45.2% of users of tablets/capsules experienced GIT complications compared to 21.1% of those who took injections (p=.046) (Table 4). 

**Table 4:** Factors associated with NSAID use-related complications among the population with GIT complications, Aseer region, Saudi Arabia p-value: Pearson's χ2 test; ^$^Exact probability test; *p < 0.05 (significant) NSAID: non-steroidal anti-inflammatory drugs; GIT: gastrointestinal tract

Factors	Had GIT complications due to NSAIDs?				p-value
	Yes		No		
	No	%	No	%	
Age in years					.326
20-30	9	42.9%	12	57.1%	
30-40	17	48.6%	18	51.4%	
40-50	10	43.5%	13	56.5%	
> 50	6	25.0%	18	75.0%	
Gender					.427
Male	30	43.5%	39	56.5%	
Female	12	35.3%	22	64.7%	
Smoking					.049^$^
Current smoker	14	51.9%	13	48.1%	
Ex-smoker	3	75.0%	1	25.0%	
Non-smoker	25	34.7%	47	65.3%	
Time to have NSAIDs					.633
After meals	25	41.0%	36	59.0%	
Before meals	12	46.2%	14	53.8%	
Any time	5	31.3%	11	68.8%	
Frequency of taking NSAIDs / day					.525^$^
One time	16	34.8%	30	65.2%	
Two times	16	47.1%	18	52.9%	
Three times	8	50.0%	8	50.0%	
4 / more times	2	28.6%	5	71.4%	
Formula using NSAIDs					.046*
Tablet / capsule	38	45.2%	46	54.8%	
Injection	4	21.1%	15	78.9%	
Had chronic health problems					.876
Yes	20	40.0%	30	60.0%	
No	22	41.5%	31	58.5%	
Use other drugs?					.298
Yes	18	47.4%	20	52.6%	
No	24	36.9%	41	63.1%	

## Discussion

Nearly 30% to 50% of NSAID users have endoscopic lesions including subepithelial hemorrhages, erosions, and ulcerations. Most lesions sited in the gastric antrum, and frequently without clinical manifestations. Commonly, these lesions have no clinical implication and spontaneously resolve with long-lasting use [10,11]. The onset of these symptoms appears to differ based on the type of NSAID. A systematic review of the accessible trails from the Cochrane Collaboration concluded that COX-2 selective inhibitor (celecoxib) was linked with fewer symptomatic ulcers, endoscopically detected ulcers, and discontinuations for GI adverse events compared with NSAIDs [12]. Inappropriately, these symptoms cannot predict the associated mucosal injury. About 50% of patients with symptoms showed normal mucosa; though more than half of users with severe peptic ulcer complications had no previous warning symptoms [13,14].

The current study aimed to understand the use of NSAIDs and the risk of gastrointestinal complications related to the use of NSAIDs among the population in the Aseer region, southern Saudi Arabia. Regarding NSAID use, the current study showed that about half of the participants used NSAIDs. As for the pattern of use, more than two-thirds reported using the drugs with pain. NSAIDs were prescribed by physicians among more than two-thirds of the users and by pharmacists among 12.6% of them. More than half of the NSAID users had the drugs after means while one-fourth of them had before meals. As for the frequency of taking NSAIDs/day, it was taken once daily among 46 (44.7%) and twice-daily among 34 (33%). A total of 84 (81.6%) had the drugs as tablets/capsules. NSAID use was significantly associated with old age and those with chronic health problems as they may experience pain and other disease-related consequences that need analgesics. Also, the older population is more diagnosed with rheumatoid disorders which include the use of NSAIDs for symptomatic treatment. The prevalence of NSAID use in patients over 65 years old is as high as 96% in the general practice setting [15,16] About 7.3% of elderly patients over 60 years old filled at least one NSAID prescription in one year period [17]. Motola D et al. in Italy reported that 65% general population had at least one drug in the previous week and, among them, 35% used NSAIDs [18]. Of the NSAID users, 20% were ≥65 years of age and 18% were chronic users. The older age groups showed an increased risk of chronic NSAID use. Among NSAIDs, the main reasons for NSAID use, as reported by subjects, were: headache (25%), osteoarticular pain (19%), unspecified pain (15%), and osteoarthrosis (9%). More than 50% of all the NSAIDs were prescribed by physicians (general practitioners, specialists, hospital-physician), whereas about 44% were taken as a self-treatment or following the advice of a pharmacist, relative/friend. In Saudi Arabia, Karami N et al. [19] revealed that paracetamol was the most common analgesic used as over-the-counter (OTC) (73.4%) then ibuprofen (13.1%). Nearly 94.4% of participants used one to two tablets of analgesics per day. More than half (60.7%) believed that analgesics must be taken after meals. The current study also revealed no significant association between male and female gender, same as the previous studies in Iran, but in contrast to other studies in Riyadh, Taif, Turkey, Germany, and Norway [20-24].

Regarding NSAID-related complications, the current study showed that less than half (41.8%) of the users experienced GIT complications. The most-reported complications were peptic ulcer (37.9%), GIT bleeding (5.8%), GIT erosions (4.9%), and intestinal obstruction (3.9%) while 59.2% had no complications. Complications were significantly higher among smokers (current and ex-smokers), and those who had NSAIDs as tablets, which is mostly due to direct contact with GIT mucosa and frequent use for a long time. These complications were the most reported in different literature studies among NSAID users [25-28].

Limitations

The present study has several limitations. The main limitation of this study was its retrospective nature and the lack of essential variables available in the hospital database system, such as the type of NSAID that has been used and dosing, limited information about the type of chronic illness among participants, and the small number of patients enrolled in the study, which will affect the ability to detect an association between GIT complication and NSAID use in the Aseer region.

## Conclusions

In conclusion, the current study revealed that nearly one out of two participants in the Aseer region used NSAIDs mainly for pain management. Old-aged participants with chronic health problems were the main users. Regarding high utilization rates, less than half of them developed GIT complications, mainly peptic ulcers. Concomitant smoking and NSAID use were very significant for GIT complications. These medications should be prescribed for the shortest duration possible in the lowest effective dose, and with careful surveillance to monitor GIT complications with other reported renal and cardiovascular toxicity.
